# Comparison of Hyaluronic Acid Versus Enamel Matrix Derivative in the Treatment of Intrabony Periodontal Defects: A Systematic Review and Meta-Analysis

**DOI:** 10.3290/j.ohpd.c_2808

**Published:** 2026-09-17

**Authors:** Théo Mahintach, Camille Bechina, Sofiya Galina, Assem Soueidan, Christian Verner, Xavier Struillou

**Affiliations:** a Théo Mahintach Resident, Nantes Université, CHU Nantes, Periodontology Department, F-44000 Nantes, France. Substantially contributed to the conception and design of the work, contributed to the acquisition of the data, contributed to the analysis and interpretation of the data, drafted and revised the previous versions of the manuscript, approved the final version of the manuscript and is accountable for all aspects of the work.; b Camille Bechina Assistant Professor, CHU Nantes, Nantes Université, Periodontology Department, UIC Odontology 11, Nantes, France; Nantes Université, CHU Nantes, INSERM, Regenerative Medicine and Skeleton, RMeS, Nantes, France. Contributed to the acquisition of the data and revised the previous versions of the manuscript, approved the final version of the manuscript and is accountable for all aspects of the work.; c Sofiya Galina Resident, Nantes Université, CHU Nantes, Periodontology Department, F-44000 Nantes, France. Contributed to the acquisition of the data, contributed to the analysis and interpretation of the data, and revised the previous versions of the manuscript; approved the final version of the manuscript and is accountable for all aspects of the work.; d; e Assem Soueidan Professor, CHU Nantes, Nantes Université, Periodontology Department, UIC Odontology 11, Nantes, France; Nantes Université, CHU Nantes, INSERM, Regenerative Medicine and Skeleton, RMeS, Nantes, France. Contributed to the analysis and interpretation of the data, revised the previous versions of the manuscript, approved the final version of the manuscript and is accountable for all aspects of the work.; f Christian Verner Associate Professor, CHU Nantes, Nantes Université, Periodontology Department, UIC Odontology 11, Nantes, France; Nantes Université, CHU Nantes, INSERM, Regenerative Medicine and Skeleton, RMeS, Nantes, France. Substantially contributed to the conception and design of the work, revised the previous versions of the manuscript, approved the final version of the manuscript and is accountable for all aspects of the work.; g Xavier Struillou Associate Professor, CHU Nantes, Nantes Université, Periodontology Department, UIC Odontology 11, Nantes, France; Nantes Université, CHU Nantes, INSERM, Regenerative Medicine and Skeleton, RMeS, Nantes, France. Substantially contributed to the conception and design of the work, drafted and revised the previous versions of the manuscript, approved the final version of the manuscript and is accountable for all aspects of the work.

**Keywords:** enamel matrix derivative, hyaluronic acid, meta-analysis, periodontal regeneration, systematic review

## Abstract

**Purpose:**

Evaluate the potential non-inferiority of hyaluronic acid (HA) when compared to enamel matrix derivative (EMD) for the surgical management of intrabony defects.

**Methods and Materials:**

A systematic search was performed in PubMed, ScienceDirect, Scopus, Google Scholar, LILACS, following PRISMA guidelines. Weighted mean differences (WMD) and 95% confidence intervals (CI) between HA and EMD were estimated using a random-effect model for clinical attachment level (CAL gain, primary outcome), probing pocket depth (PPD), and gingival recession (REC). A non-inferiority margin of 0.5 mm was defined for CAL. In order to minimise bias, only randomised clinical studies (RCTs) were selected.

**Results:**

Across 117 articles, 3 RCTs were included. HA achieved clinical non-inferiority in CAL gain versus EMD (WMD = 0.51 mm, CI [–0.08; 1.11] at 12 months, WMD = 0.53 mm). Regarding PPD, the data do not support the non-inferiority hypothesis in comparison to EMD at 12 months (PPD: WMD = 0.71 mm, CI [–0.11; 1,54], I^2^=72.5%). For gingival recession, HA demonstrated clinical non-inferiority at 12 months (WMD = -0.4 mm, CI [–0.79; –0.01], I^2^ = 0).

**Conclusion:**

Within the limits, the present data indicate that HA could be an alternative to EMD in intrabony defects. Further RCTs are required to consolidate these preliminary results.

Prospero registration number: CRD420261301986

Periodontitis is a widespread inflammatory disease caused by a dysbiosis in the bacterial biofilm,^9,18
^ ranking among the six most common diseases in modern society.^30,72
^ Leading, if untreated, to tooth loss, its clinical manifestations are clinical attachment loss (with a mean of 0.5 mm annual loss^31^), periodontal pocket formation and radiographically detectable bone loss.^47^ Bone defects, when associated with periodontal pockets, represent a risk for further progression.^48^ They are described as alveolar osseous defects with a base located apical to the interdental alveolar crest, and enclosed by bony walls (from one to three).^14^ The European Federation of Periodontology (EFP) recommends the use of a surgical access for residual probing pocket depth (PPD) ≥ 6 mm, with periodontal regenerative therapy for bone defects > 3 mm.^62^ In a recent meta-analysis, periodontal regenerative therapy presents better patient-related outcomes and cost-effectiveness when compared to implant placement with fixed prosthesis or open flap debridement.^7^ Among biomaterials, periodontal regenerative therapies (guided or induced) have shown statistical differences when compared to open flap debridement alone.^54,68
^ Using guided periodontal regeneration, the use of biomaterials such as allogeneic graft, demineralised freeze-dried bone allograft or hydroxyapatite for the treatment of intrabony periodontal defect showed a CAL gain of 0.44 to 1.2 mm, with a follow-up period ranging from 6 months to a year. A recent study showed that after 7 years post-surgery, 44% of the sites presented a CAL gain > 3 mm, with PPD < 4 mm.^4^ It is important to mention that periodontal supportive care (step 4 of the EFP guidelines) is crucial to maintain long-term favourable outcomes, as highlighted by Rattu et al^53^ in a meta-analysis including articles with at least 10 years of follow-up.

Periodontal regeneration is defined as the healing *ad integrum* of the periodontal attachment.^81^ Its mechanism is complex and was reported by Melcher in 1976.^34^ This *in vitro* study showed that the proliferation of periodontal ligament cells inhibited osteoblastic differentiation, which could explain the mechanism for the stability of the periodontal ligament.

Regarding cellular colonisation and its impact on periodontal healing, Karring et al experimented on dog premolars, with a ligature system to induce periodontitis. They defined several mechanisms, such as resorption (by ankylosis), reattachment with a long junctional epithelium and periodontal regeneration (if the periodontal ligament is preserved).^28^ This specific need for periodontal ligament stem cells can be answered by excluding undesired cells.^51,73
^


To optimise periodontal regeneration, primary stability of the wound is mandatory, as found by Linghorne et al in 1950 during their experiments on dogs.^33^ This primary stability can lead to a better adhesion of the blood clot with a higher quantity of fibronectin, as shown by Polson in 1983.^52^


Therefore, optimal wound healing and periodontal regeneration need to follow the PASS acronym (although used historically for predictable bone regeneration): primary stability of the wound, angiogenesis, stabilisation of the blood clot, and space maintenance.^79^


Enamel matrix derivatives (EMD), commercialised as EMDOGAIN®, is an injectable biomaterial containing derivatives of enamel matrix proteins from porcine animals,^19^ often associated with a preparative gel of EDTA at 24%.^61^ It has shown clinical and radiological effects, associated or not with bone-derived grafts, that led it to be one of the standards for periodontal regeneration.^37,38,55
^ Compared with guided tissue regeneration (with a membrane), it showed fewer complications with similar clinical results.^63,75
^ However, its composition is unclear, as well as its mechanism of action: in mice, it has shown a similar effect on cells without amelogenin receptors,^22^ but also *in vitro* via the stimulation of the transforming growth factor-β receptor I pathway, either on adipocytes^16^ or in human periodontal ligament fibroblasts.^67^ Moreover, a study by Sculean et al showed that on 24 intrabony defects, the addition of EDTA didn’t yield a statistical difference with a 1-year follow-up.^64^ Therefore, EMD can intervene in the recruitment of periodontal ligament stem cells, leading to periodontal regeneration, but its detailed mechanism remains unclear.

Due to its porcine origin, Emdogain® can also show some limitations regarding cultural and religious beliefs.

Hyaluronic acid (HA) as an adjunct to periodontal regeneration is a novel biomaterial, first evaluated in 1999 and gaining interest since,^39^ with early clinical cases for the treatment of intrabony defects in 2009.^3,77
^ It is an anionic, linear, non-sulfated glycosaminoglycan structured biomolecular, and comprises the extracellular matrix of nearly all organs. In the periodontal attachment, it is synthesised by fibroblasts, periodontal ligament cells, cementoblasts and osteoblasts,^27^ and therefore presents a high biocompatibility.^41^ Its molecular weight is classified as high molecular weight (with up to 25000 disaccharide units), while during its degradation it can be classified as a low-molecular-weight biomolecule.^50^ In several studies, HA showed some interesting properties for periodontal regeneration: clot stabilisation with the HA acting as a matrix for the fibrin,^40^ anti-oedematous with its hygroscopic nature,^13^ anti-inflammatory effects with CD44,^60^ angiogenetic properties in its low molecular weight form^56^ and bacteriostatic effects.^5^ Following the PASS principles of periodontal surgery, HA seems to intervene in the stability of the blood clot and angiogenesis.^79^ Several systematic reviews showed a clinical and radiological difference in favour of hyaluronic acid, when compared to open flap debridement, including in the management of intrabony defects.^10,44,59
^ HA, as a biomaterial, has a precise and known molecular composition, and can be industrially synthesised, limiting the usage of animals for its production.^25,65
^


However, no previous reviews investigated its clinical implications in the surgical management of intrabony periodontal defects, when compared with the periodontal regenerative standard that is EMD.

The aim of this systematic review is to compare HA to EMD in the surgical treatment of periodontal intrabony defects.

## Methods and Materials

A systematic review of the literature following the preferred reporting items for systematic reviews and meta-analyses (PRISMA) guidelines was conducted.^46^ The protocol was registered in the PROSPERO database under number CRD420261301986.

### Eligibility Criteria According to PICO Framework

The PICO criteria (population [P], intervention[I], comparison [C], outcome [O]) for this study were: (1) P – Adult patients suffering from intrabony periodontal defects, in need of periodontal regeneration; (2) I – Intervention with the sole use of HA as a biomaterial used during the surgery; (3) C – Compared with patients exposed to EMD; (4) O – Assessment of the clinical and radiological parameters.

#### Inclusion criteria

Inclusion criteria were: 1) Full-text article available in English; 2) Randomised Controlled Trials among adult human subjects; 3) The article must treat the role of HA and compare it to EMD in the treatment of intrabony defects; 4) Article published after the year 2010 (included).

#### Exclusion criteria

The following exclusion criteria were applied: 1) Case report, or case series, expert opinion, mechanical theoretical studies, experimental studies (animal studies), *in vitro* studies, non-randomised controlled trial, literature reviews or meta-analyses, conference resume, non-scientific press article; 2) Studies that were not retrievable or in another language than English; 3) Studies that did not compare HA and EMD; 4) Studies that used external factors or therapies on either the control or the experimental site.

The use of external factors is defined as a variation in the surgical protocol in a specific group that is not justified by the biomaterial preparation and/or application.

### Search Strategy

The following electronic databases were searched to identify relevant studies from the start of each database through June 2026: Medline, Scopus, Science Direct, LILACS, Cochrane Library and Google Scholar. In addition, the reference lists of included articles were also screened.

Regarding the search strategy, the following MESH terms were included: ‘hyaluronic acid’[MeSH Terms], or ‘enamel proteins, dental’[MeSH Terms], or ‘guided tissue regeneration, periodontal’[MeSH Terms].

The search strategy included the following terms : (‘hyaluronic acid’ OR ‘acid, hyaluronic’) AND (‘enamel matrix derivatives’ OR ‘emdogain’) AND (‘intrabony periodontal defect’ OR ‘periodontal regeneration’ OR ‘guided tissue regeneration, periodontal’).

Several filters have been added to Google Scholar, such as publication year (from 2010 to 2026), the presence of the terms in the abstract, introduction or title. Literature reviews, case reports and conference articles were excluded using a filter. The detailed search strategies can be found in the Appendix.

### Study Selection

All identified studies were screened per database and were reviewed in two stages.

After removing duplicates resulting from the search, the first selection round was made by two independent reviewers (TM and XS) who read the titles and abstracts. Then, after reading the full text, a second selection was performed by two independent reviewers (TM and XS). In cases of disagreement, a third reviewer was involved (CV). The initial interrater agreement between TM and XS for full-text screening was 100%.

### Data Collection

Data extraction was independently performed by two reviewers (TM and XS).

Data were collected on the following: type of study, date of the study, type of population, type of therapy, main outcome, secondary outcomes, number of groups, sample size per group, randomisation and its method, blinding, measuring tools (i.e. clinical and radiological parameters such as probing of pocket depth, clinical attach loss, gingival recession, bleeding on probing, full-mouth plaque score), date of measurement, statistical tests, statistical values (or *p* value), main outcome, and secondary outcomes. Considering the outcomes, the following data were extracted: probing pocket depth, clinical attachment loss, gingival recession, bleeding on probing, suppuration on probing, marginal bone loss, percentage of the defect filled.

For patients treated with EMD, specific parameters were collected: type of EMD (to ensure that only surgical EMD was used), preparation protocol, root conditioning protocol.

For the HA, specific parameters were collected: concentration, degree of reticulation, root preparation protocol.

Regarding the surgery, anaesthetic protocol, flap access design, type of sutures (material, protocol), postoperative and preoperative medication were collected.

### Analyses

Descriptive analyses were performed with R Software (version 4.5.3).

Meta-analyses using weighted mean differences (WMDs) and bilateral confidence intervals at 95%, as recommended by the CONSORT Guidelines, were performed. Given the potential heterogeneity linked to various surgical protocols, a random-effects model was systematically applied using the restricted maximum likelihood method. Given the different follow-up periods, subgroup explorative meta-analyses were performed at different time points.

Heterogeneity between studies was evaluated with Cochran’s Q and quantified by the I^2^ coefficient. Between-studies variance was estimated with τ^2^.

Secondary outcomes (such as bleeding on probing (BOP), PPD, gingival recession (REC), defect depth) were analysed as explorative outcomes. The null hypothesis was: ‘HA presents strictly inferior results to EMD in CAL gain, with a margin of 0.5 mm’.

#### Quality of the studies

The level of evidence was assessed via the ‘Oxford 2011 Levels of Evidence’ classification.^42^


The risk of bias and quality of study were assessed depending on the type of study and according to Cochrane’s recommendations.^24^ Therefore, the Rob2 for parallel-arm randomised clinical trials was used.

## Results

### Selected Articles

A total of 117 articles were screened (20 from PubMed, 12 from ScienceDirect, 37 from Scopus, 25 from Google Scholar, 9 from LILACS, 9 from the Cochrane Library and 5 through manual research), of which 18 duplicates were removed. The flow chart is presented in Figure 1.

**Fig 1 Fig1:**
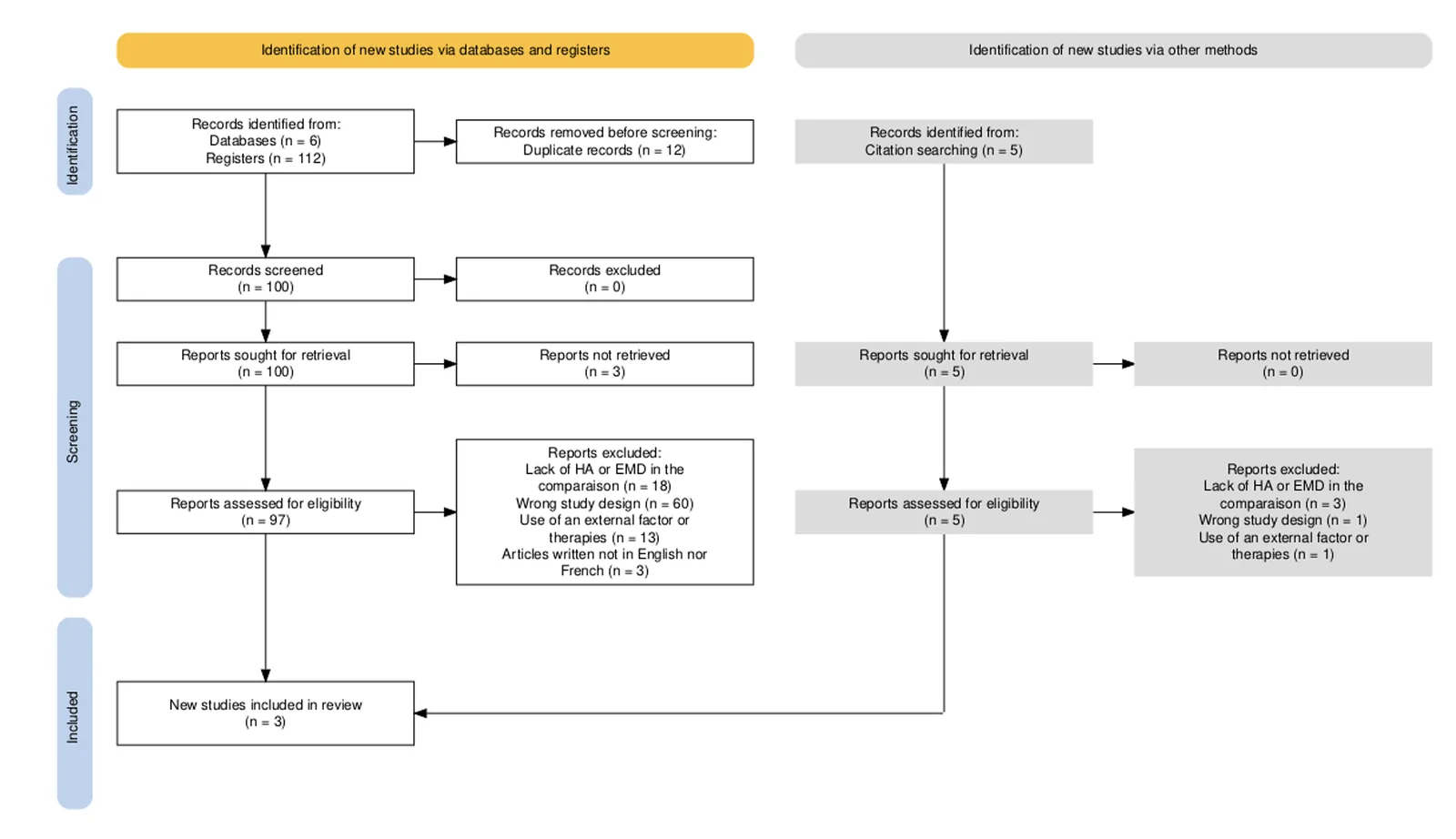
PRISMA flow chart of the meta-analysis.

Three randomised controlled trials were included in the review.^49,78,57
^


#### Characteristics of the included studies

139 intrabony defects were treated in the 3 included studies, across 139 adult patients. Prior to the surgery, all patients benefited from a non-surgical periodontal treatment for 6^78^ to 12 weeks in advance.^49,57
^


All the articles included intrabony defects with a pre-surgical depth > 3 mm, with at least two walls. The presence of buccal or lingual dehiscence was not an exclusion criterion. All surgeries were performed under local anaesthesia, with minimal flap designs (either mucoperiosteal flap,^78^ mini-invasive surgery technique^57^ or single flap access^49^). After degranulation, root scaling was performed with a hybrid method (mechanical and ultrasonic techniques). Only non-resorbable tension-free sutures were performed and removed 2 weeks post-surgery. Two studies had postoperative antibiotics^49,57
^ and 3 had oral rinses of chlorhexidine.^49,57,78
^


Regarding the intrabony defects, only one defect was included per patient. The surgical sites were not specified and included, therefore, all tooth types and localisation. One study^49^ didn’t include one-wall intrabony defects, while the others indicated that a wall couldn’t present a dehiscence of more than 2 mm. However, in all three studies, the statistical analyses performed by the authors indicated the lack of statistical difference between study groups.

All studies used the same biomaterial: Emdogain® for EMD and HyaDent BG® for HA. The latter is composed of 2 mg natural hyaluronic acid, 16 mg cross-linked reticulated hyaluronic acid, 6.9 mg of sodium chloride, and 1 ml of water.

Follow-up ranges from 6 to 24 months, with periodontal maintenance every 3 to 6 months.

Clinical measurements were made using a mechanical probe (PCP-UNC 15; HuFriedy Manufacturing, Chicago, IL, USA), and radiological measurements were based on retro-alveolar radiographs with parallel techniques.

One study didn’t report the radiographic parameters,^49^ as it lacked a standardised realisation, according to the authors.

All the characteristics and outcomes are reported in Table 1.

**Table 1 Table1:** Characteristics of the included studies: number of patients, surgical protocol, postoperative treatment, Type of HA, methodology of measurements and outcomes. The table shows different study protocols, with similarities in the clinical measurements (periodontal probe PCP-UNC 15), and the same hyaluronic acid (HyaDENT BG, Bioscience)

Study, date	Number of patients (experimental/control)	Type of flap	Degranulation	Sutures	Postoperative treatment (D = day)	HA	Main outcome	Secondary outcomes	Methods of measurements (product)	Time of measurements (months)
Pilloni et al, 2021	32 (16/16)	Single Flap Access (Buccal) +/– vertical incision	Gracey curettes + Ultransonic scalers	6-0 non-resorbable monofilament Horizontal internal mattress suture	Amoxicillin 2 g/D for 6 D CHX oral rinse 0.2% 2*/D for 14D Ibuprofen 1200 mg/D for 3D	HyaDENT BG, Bioscience (2 mg natural hyaluronic acid, 16 mg cross-linked reticulated hyaluronic acid, 6.9 mg sodium-chloride, 1 ml water)	CAL	Clinical: PPD, REC, BOP, FMPS, FMBS, EHI	Clinical: Periodontal probe (PCP-UNC 15; HuFriedy Manufacturing, Chicago, IL, USA)	12, 18, 24
Vela et al, 2024	54 (27/27)	Muco-periosteal flap +/– vertical incision	Gracey curettes + Ultransonic scalers + Airflow	6-0 non-resorbable monofilament	CHX oral rinse 0.2% 2*/D for 2 weeks Ibuprofen 1200 mg/D for 3D	HyaDENT BG, Bioscience (2 mg natural hyaluronic acid, 16 mg cross-linked reticulated hyaluronic acid, 6.9 mg sodium-chloride, 1 ml water)	CAL	Clinical: PPD, REC, BOP, FMPS, FMBS, EHI Radiological: defect depth, defect width	Clinical: Periodontal probe (PCP-UNC 15; HuFriedy Manufacturing, Chicago, IL, USA) Radiological: parallel technique (CliniView Imaging Software, Instrumentarium Dental/PaloDEx Group; Tuusula, Finland)	6
Rodriguez et al, 2025	53 (27/26)	M-MIST or MIST	Gracey curettes + Ultransonic scalers	6-0 or 7-0 e-PTFE, internal modified mattress	Amoxicillin 1.5 g/D for 7 D CHX oral rinse 0.12% 2*/D for 28D	HyaDENT BG, Bioscience (2 mg natural hyaluronic acid, 1 6 mg cross-linked reticulated hyaluronic acid, 6.9 mg sodium-chloride, 1 ml water)	CAL	Clinical: PPD, BOP, REC, FMBS, FMPS, Dental mobility, Radiological: Defect Depth, % of periodontal regeneration, % of defect fill	Clinical: Periodontal probe (PCP-UNC 15; HuFriedy Manufacturing, Chicago, IL, USA) Radiological: Parallel technique (VistaScan, Dürr Dental, Germany)	6, 12, 18
MIST: minimally invasive surgical technique, M-MIST: modified minimally invasive surgical technique, CHX: chlorhexidine, CAL: clinical attachment loss, PPD: probing pocket depth, REC: recession, BOP: bleeding on probing, FMPS: full-mouth plaque score, FMBS: full-mouth bleeding score, EHI: early healing index.										

### Meta-Analyses

#### Primary outcome

The meta-analysis for the CAL gain is reported in Figure 2. At 6 months, non-inferiority of HA compared to EMD could not be achieved, with a tendency to similar results (WMD = 0.01 mm, CI [–0.66; 0.67]). In contrast, at 12 and 18 months, the meta-analysis shows non-inferiority of HA with EMD (WMD = 0.51 mm, CI [–0.08; 1.11] at 12 months, WMD = 0.53 mm, CI [–0.17; 1.24] at 18 months). Heterogeneity remained low across all time points (I^2^ ranging from 0% to 28.9%), and no statistically significant differences were found between subgroups (*p* = 0.52).

**Fig 2 Fig2:**
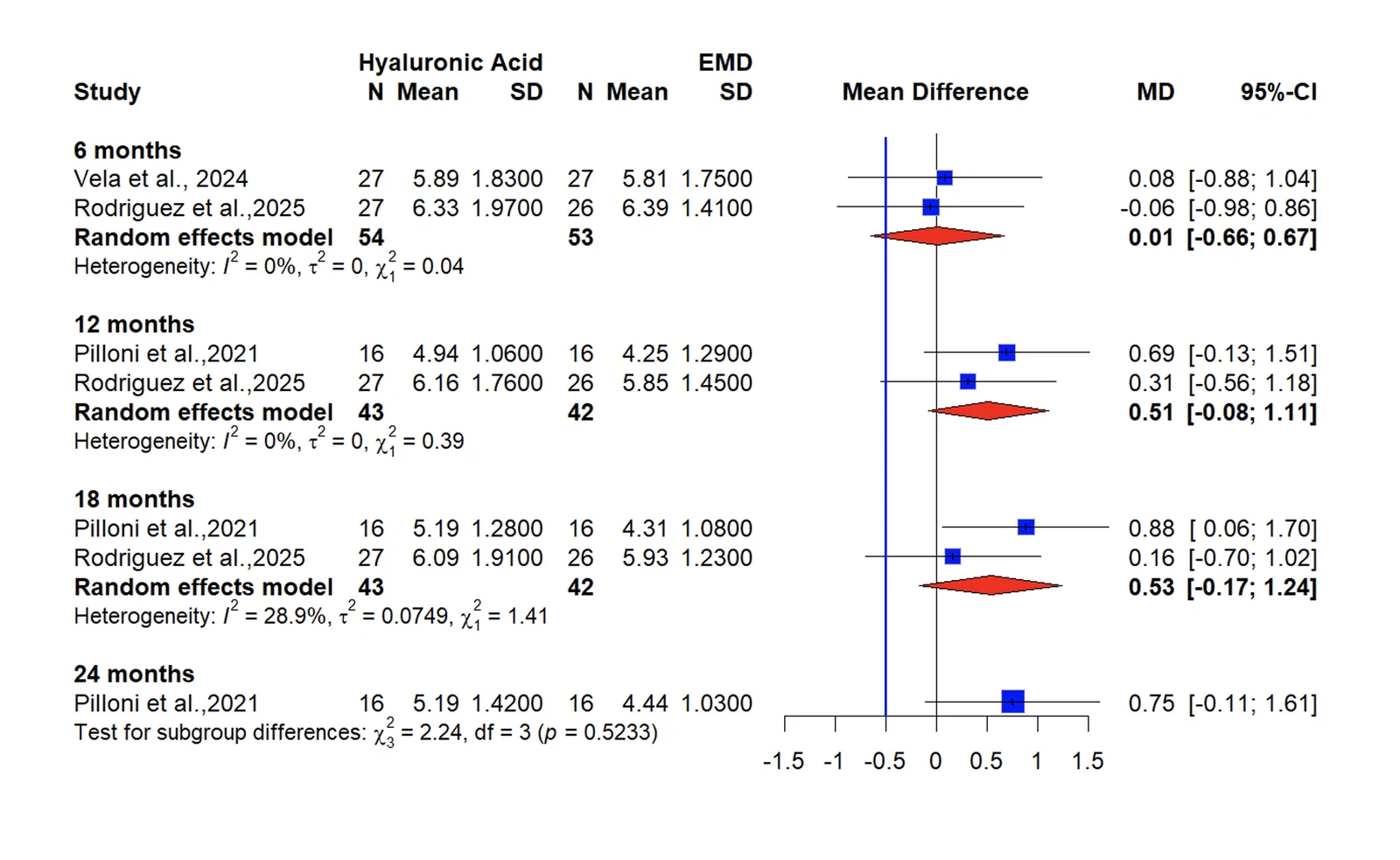
Forest plot of HA versus EMD in CAL gain (mm) after intrabony defect surgery. Values on the right of the blue line at –0.5 (non-inferiority hypothesis) favour HA (i.e. more CAL gain in HA than EMD).

#### Secondary outcomes

The meta-analysis for the PPD is reported in Figure 3. At 6 months, the difference between HA and EMD groups was WMD = 0.03 mm; CI [–0.47; 0.53], I^2^ = 0%. A clinical trend favouring EMD emerged at 12 months (WMD = 0.71 mm) and 18 months (WMD = 0.7 mm), yet it didn’t reach statical significant intervals (CI [–0.11; 1.54] at 12 months, CI [0.62; 1.80] at 18 months), and a high heterogeneity at these time points was observed (I^2^ > 70%), with a significant subgroup differences (*p* = 0.02). Therefore, the current data cannot be interpreted in favour of either group, due to high heterogeneity.

**Fig 3 Fig3:**
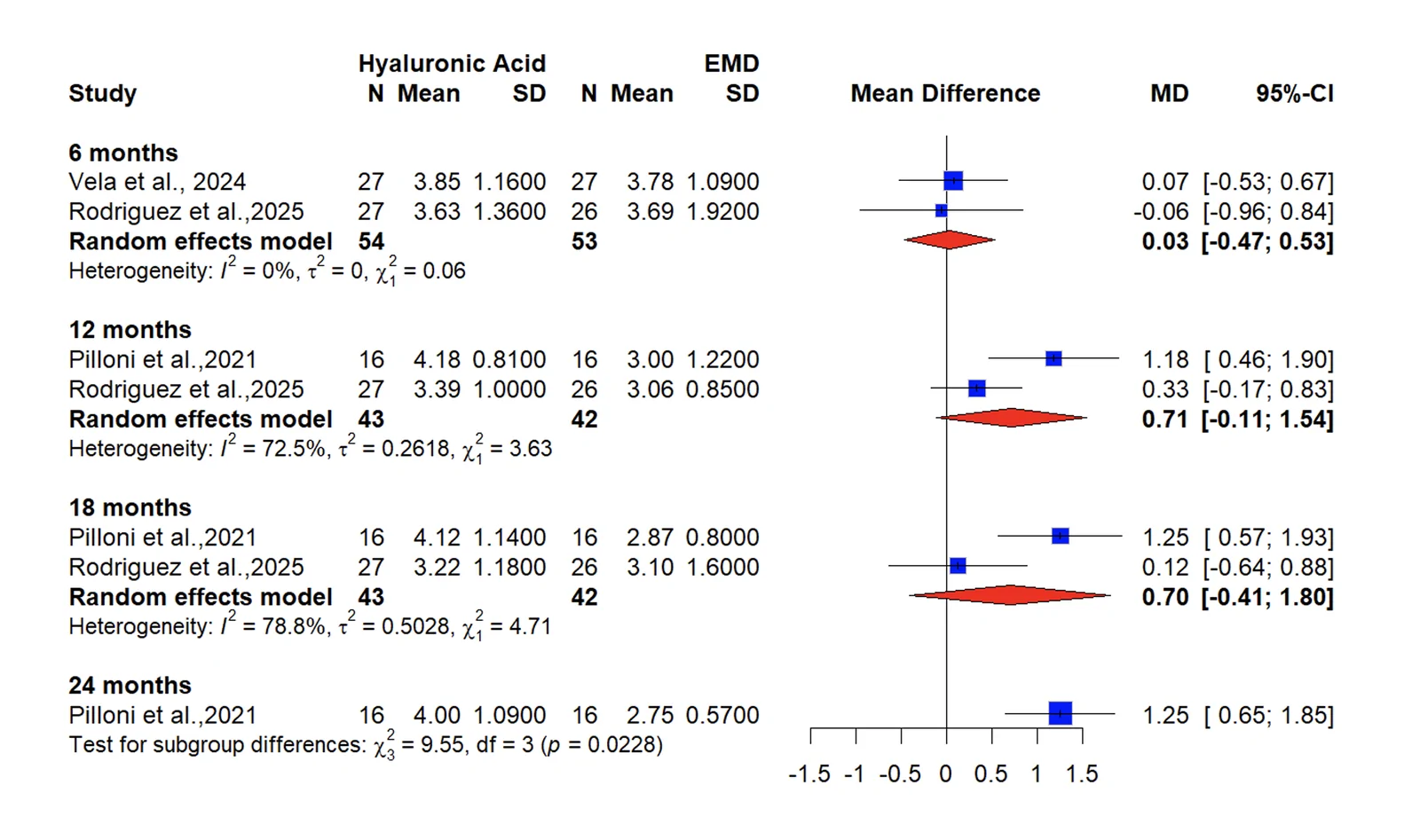
Forest plot of HA versus EMD in PPD (mm) after intrabony defect surgery. Values on the right of the reference line favour EMD (i.e. more PPD in HA than EMD).

The meta-analysis for the REC is reported in Figure 4. It reveals at 12 months a non-inferiority of HA when compared to EMD (WMD of –0.4 mm for HA, CI [–0.79; –0.01]), with no heterogeneity. Throughout the other time points, HA tends to show similar results in post-surgical REC (ranging from –1 mm of REC at 24 months to +0.55 mm at 6 months).

**Fig 4 Fig4:**
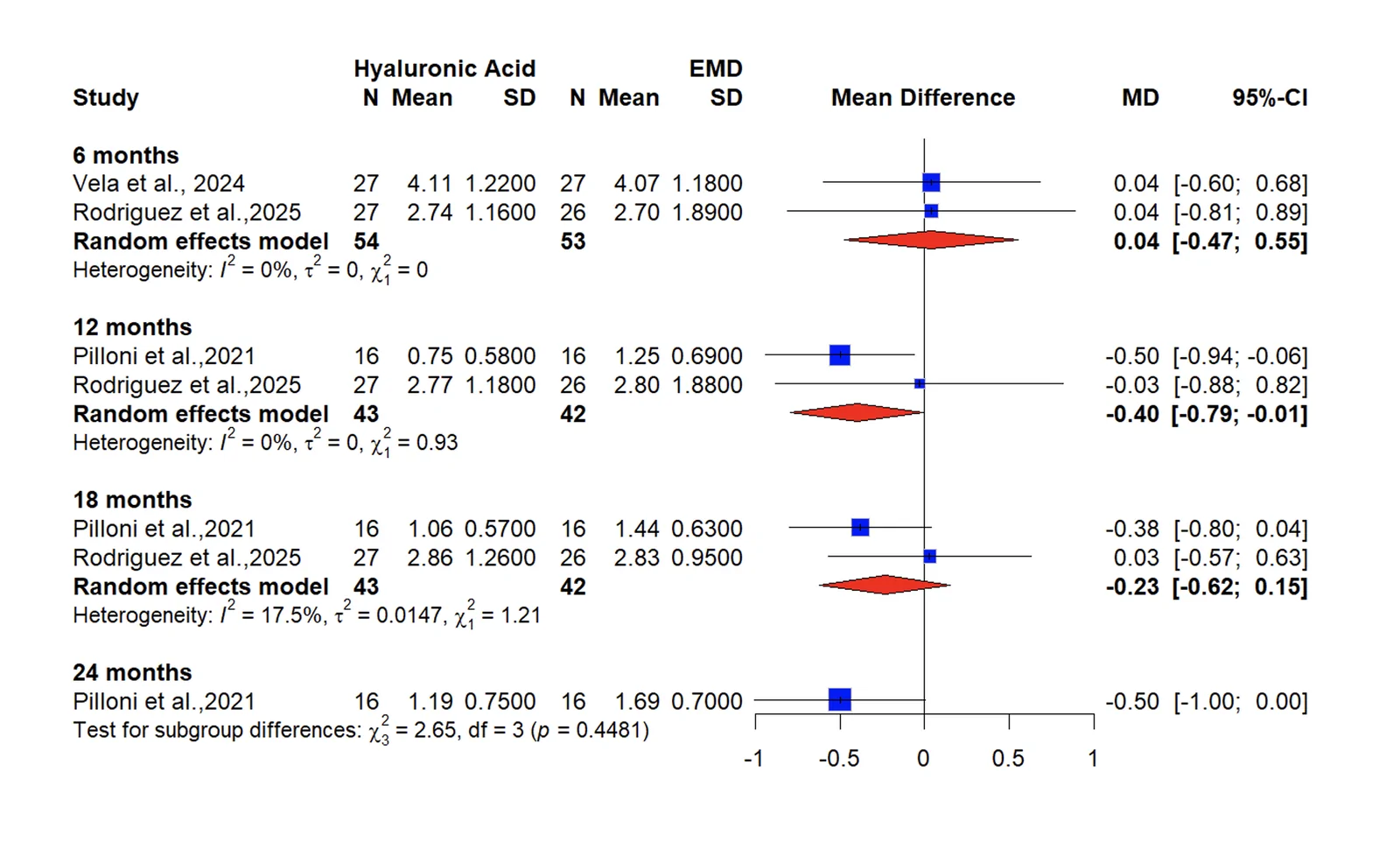
Forest plot of HA versus EMD in REC (mm) after intrabony defect surgery. Values on the right of the reference line favour HA (i.e less REC in HA than EMD).

The meta-analyses for the BOP and the defect depth are reported respectively in Figures 5 and 6. Regarding BOP, HA did not present non-inferiority results to EMD, with a tendency towards less BOP (WMD = –0.08%, CI [–0.24; 0.08] at 12 months, WMD = –0.13%, CI [–0.32; 0.05] at 18 months). Regarding defect depth, HA did not present non-inferiority results to EMD (WMD = +0.12 mm, CI [–0.19; 0.43]).

**Fig 5 Fig5:**
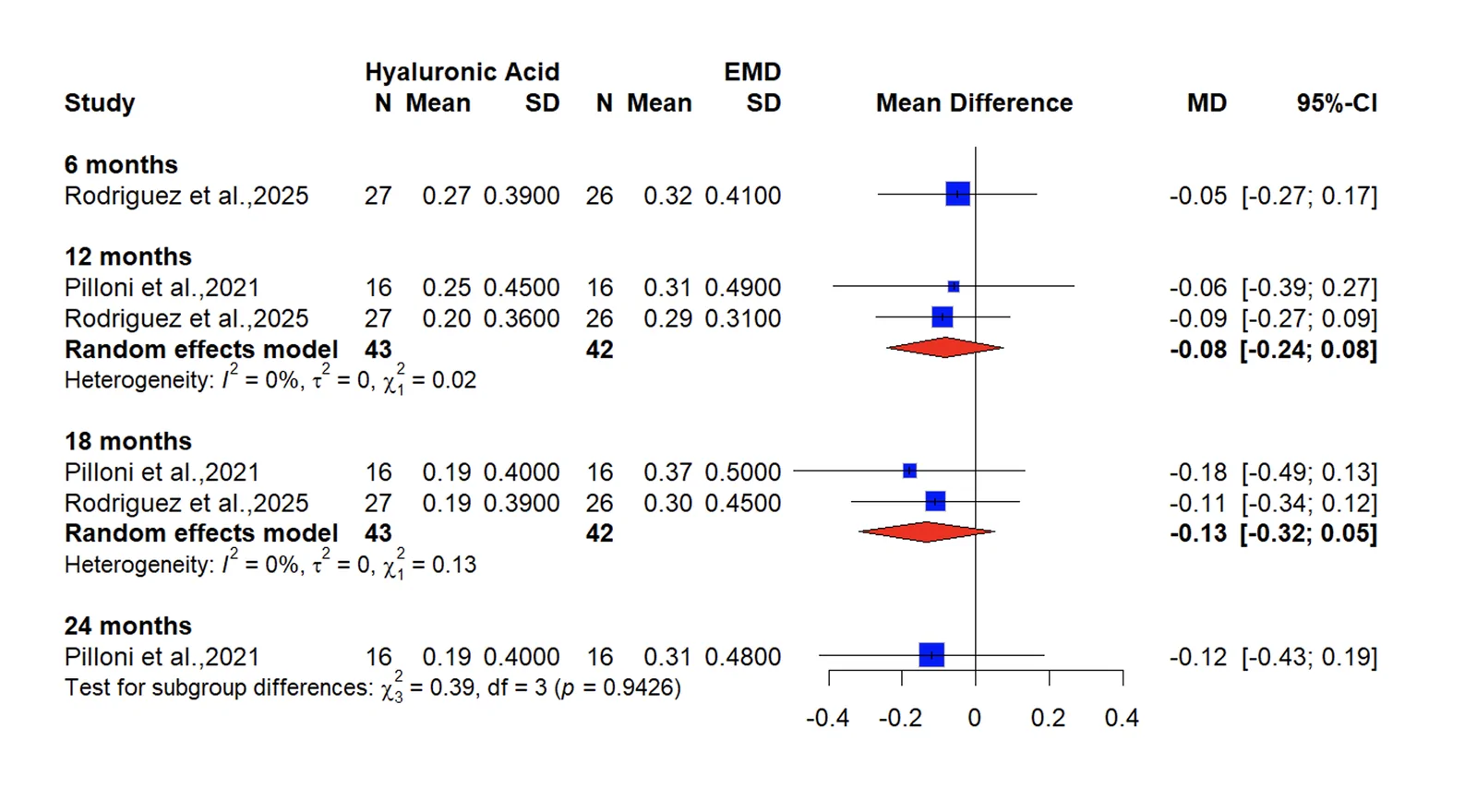
Forest plot of HA versus EMD in BOP (%) after intrabony defect surgery. Values on the right of the reference line favour HA (i.e. less BOP in HA than EMD).

**Fig 6 Fig6:**
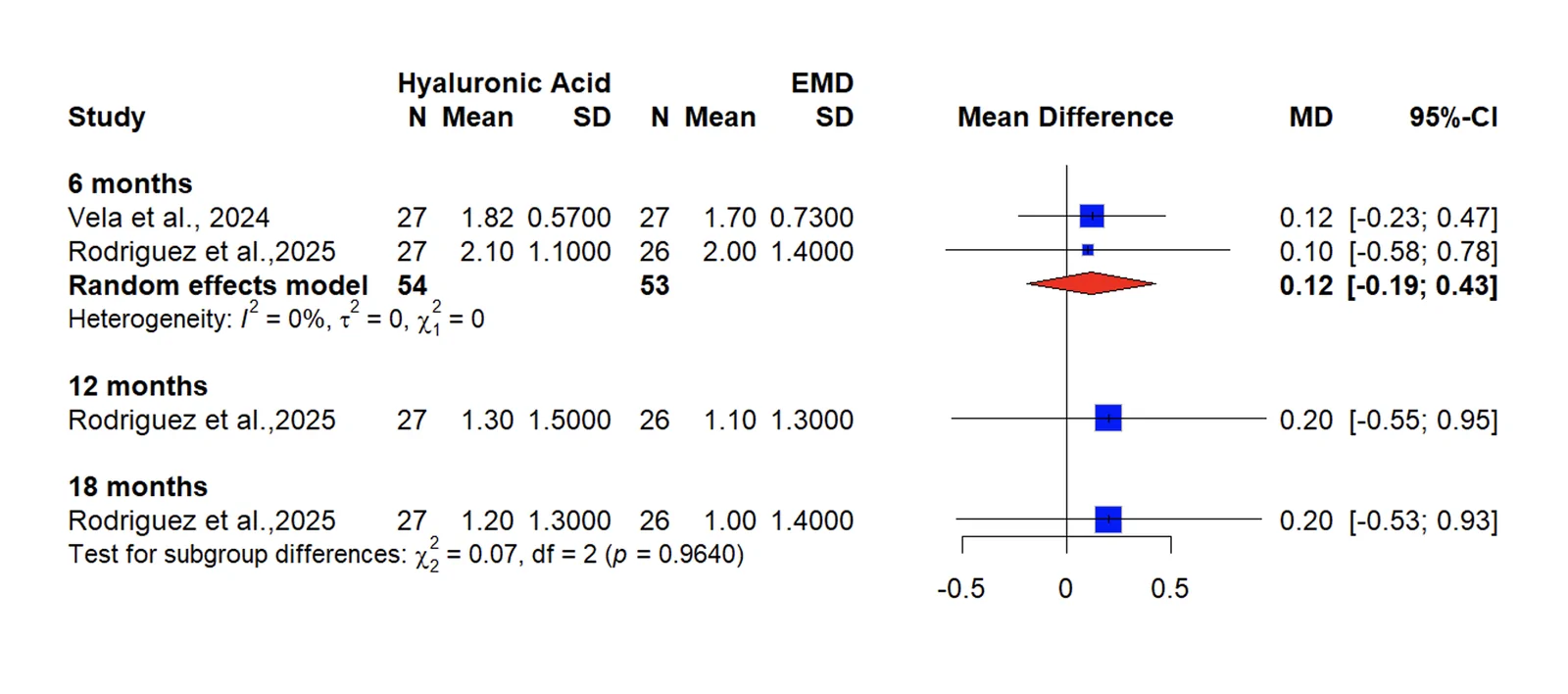
Forest plot of HA versus EMD in radiological Defect Depth (mm) after intrabony defect surgery. Values on the right of the reference line favour EMD (i.e less depth in the EMD group versus HA).

#### Assessment of biases

Three studies were analysed following Cochrane’s Handbook recommendation.^24^ All the bias evaluations are listed in Table 2 with a low^49,57,78
^ risk of bias across all articles, with a 100% interrater agreement.

**Table 2 Table2:** Assessment of the risk of bias with Cochrane’s Handbook and Rob2 Tool. The use of ‘+’ for each column reflects a low risk-of-bias judgement for each sub-category and ‘?’ reflects some concerns

Study, date	OECBM level of evidence	Randomisation process	Effect of assignment to intervention	Effect of adhering to intervention	Missing outcome data	Measurement of the outcome	Selection of the reported results	Average bias
Pilloni et al, 2021	2	+	+	+	?	+	+	Low
Vela et al, 2024	2	+	+	+	+	?	+	Low
Rodriguez et al, 2025	2	+	+	+	+	+	+	Low


## Discussion

### Principal Findings

Our meta-analysis demonstrated non-inferiority of HA when compared with EMD for the CAL gain in the surgical treatment of intrabony defects, with a margin of 0.5 mm, at 12 and 18 months. To our knowledge, at the time of publication, no literature reviews nor meta-analyses compared HA to EMD in the surgical management of intrabony defects. HA has already been studied as an adjunct for non-surgical periodontal therapy without showing statistical significance towards CAL gain,^8^ and in the surgical management of intrabony defects, with statistical significance in CAL gain when compared to open flap debridement alone.^45^


The choice of a non-inferiority margin of 0.5 mm was motivated by several arguments, one being that this margin was used in the sample study calculation of all the included articles, and is used by the European Federation of Periodontology as a clinical relevance margin.^62^ The first randomised controlled trial^49^ based its calculations on a clinical trial of 1997 in which Emdogain® was compared to open flap debridement for intrabony periodontal defects.^23^ The 0.5 mm margin is also associated with the computed standard deviation found by Goodson in 1986, on 48,064 sites.^15^


Our results towards REC show that HA could have an interaction with soft tissue management. It is interesting to note that HA, which has been evaluated as an adjunct to the coronally advanced flap, has not shown statistical significance for single recessions.^58,70
^ In a 2024 randomised controlled trial, HA showed a statistical difference in the soft tissue texture and, histologically, with a more dense collagen matrix after tunnel technique and subepithelial connective tissue graft for multiple gingival recessions.^66^ However, the biological pathways and the objectives differ widely between muco-gingival surgery and periodontal regeneration.

Several hypotheses emerge from the principal findings: Do the parameters of HA interfere with the clinical results?

In immortalised periodontal ligament cells, Frasheri et al found a statistical difference in metabolic activity with HA with weights of 75–350 kDa or 15–40 kDa. However, regarding mineralisation (evaluated with a calcium deposition assay), 15–40 kDa HA showed more calcification at 21 days, when compared to 75–350 kDa HA^12^. Another interrogation could be on the reticulation of HA and its incidence towards cellular activity. On fibroblasts, both non-cross-linked HA and cross-linked HA stimulated cell proliferation and collagen formation, although the latter induced a higher expression of mRNA coding for fibroblast-growth factor.^22^ Cross-linked HA can also be associated with photoactivators to enhance its degradation,^26^ or loaded with particles such as hydroxyapatite to enhance osteogenesis and new bone formation in a murine model.^32^ It also seems to diminish early bacterial biofilm formation (with red and orange complexes), with fewer bacteria. HA presents a short half-life in the plasma, from 2.5 to 5.5 min,^43^ and the use of cross-linking extends its presence in the cicatricial space.^17^


Concerning the EMD, another interrogation could be regarding its impact on the periodontal stem cells.^82^ EMD relies on the absorption of its active principles by the periodontal cells.^38^ There is an effect of blood contamination that may negatively influence its adsorption onto the root surfaces.^35,36
^ Several vehicles have been studied to mitigate these effects. Nowadays, propylene glycol alginate is in the composition of EMD,^20^ despite its poor structural stability.^1^


However, caution is mandatory when evaluating *in vitro* results and implying a direct extrapolation to *in vivo* results.^21^ Most of the articles work on specific cells in a 2D model, with a lack of defence metabolism, a fixed degree of oxygenation, and the unknown fate of test agents once in the culture medium. To mitigate this limitation, microphysiological systems are developed; however, their cost remains a limiting factor.

Further studies are needed to optimise the liberation of EMD and HA, while maintaining favourable conditions for periodontal regeneration.

### Limitations of the Study

Our meta-analysis suffers from several limitations, one being the small number of studies with variant follow-ups. This led to subgroup analyses with only two articles per group.

Another limit is the methodology of measurement that varied between studies, especially on the radiographical parameters (two different methodologies for the estimation of the defect depth,^57,78
^ while the third didn’t analyse radiological data due to non-standardised radiographs).^49^ The surgical protocol could also influence the results of the studies, as shown by a network meta-analysis, where a single flap approach seems to yield better CAL gain for EMD in intrabony defects, compared to minimally invasive variants.^74^


While the present article seems to demonstrate the non-inferiority of HA at 12 and 18 months, when compared to EMD in CAL gain, no conclusion can be fully drawn for the secondary outcomes (especially PPD with extremely high heterogeneity and standard deviation).

Given the small number of articles, publication bias cannot be clearly assessed.

A strength of our study is the similarity in the biomaterial for the HA group. All three RCTs used the same HA, with the same mechanical properties and molecular composition.

Precautions should be taken regarding the direct clinical application of HA in the treatment of intrabony defects, as its composition and molecular parameters lead to tissular modification.^50^ If the HA has a reticulation degree with a molecular weight over 500 kDa (high molecular weight), it becomes less resorbable^11,29
^ and presents antiangiogenic^71^ and anti-inflammatory properties.^76^ Its degradation leads to low molecular weight HA.

The lack of reticulated HA (with HA with a size inferior to 200 kDa, low molecular weight) is linked to higher angiogenesis^80^ and the recruitment of stromal cells.^69^


## Conclusion

Within the limitations of the present meta-analysis, HA presents non-inferior clinical results in CAL when compared with EMD for periodontal regeneration of intrabony defects. HA seems to be a promising alternative to EMD, with less recession and similar PPD. Histological and radiological analyses are required to evaluate the effect of HA on bone neoformation. Further multicenter, randomised, double blind, controlled clinical trials are required to confirm these results.

## References

### Appendix

**Table A1 tableA1:** Search strategies for each database – a grey literature search was also conducted. In the case of multiple search equations for a database, the number of studies identified is the number of unique studies across all equations

Database	Date of last search	Search equation	Added filters	Number of articles identified
PubMed	4-06-2026	((hyaluronic acid[MeSH Terms]) AND (dental enamel proteins[MeSH Terms])) AND (guided periodontal tissue regeneration[MeSH Terms]) ((hyaluronic acid[MeSH Terms]) AND (guided tissue regeneration, periodontal[MeSH Terms])) (hyaluronic acid[MeSH Terms]) AND (enamel proteins, dental[MeSH Terms])	For the third equation, the filter ‘Clinical trial’ was used	20
Scopus	4-06-2026	( ‘hyaluronic acid’ [MeSH Terms] ) AND ( ‘periodontal tissue regeneration’ [MeSH Terms] ) AND PUBYEAR > 2010 AND PUBYEAR < 2026 AND ( LIMIT-TO ( DOCTYPE, ‘ar’ ) )	Year of publication 2010–2026 Type of document: Article	37
ScienceDirect	4-06-2026	(‘hyaluronic acid’ AND ‘infrabony’ AND ‘enamel matrix’)	Research articles Subject: Medicine and dentistry	12
Google Scholar	11-06-2026	allintitle: hyaluronic acid periodontal regeneration	Date: 2010–2026 Language: English or French Include additional library : Nantes University Excluding citations and patents	25
LILACS	11-06-2026	(hyaluronic acid) AND (periodontal tissue regeneration) AND (enamel matrix)		9
Cochrane Library	11-06-2026	(hyaluronic acid) AND (periodontal tissue regeneration) AND (enamel matrix)	Including clinical trials protocols, excluding systematic reviews	9

